# MSTune: A Data-Driven Approach to Parameter Tuning Using Grid Search and Differential Evolution for Gas Chromatography–Mass Spectrometry-Based Compound Identification

**DOI:** 10.3390/metabo16060428

**Published:** 2026-06-18

**Authors:** Hunter Dlugas, Jing Li, Xiang Zhang, Seongho Kim

**Affiliations:** 1 Biostatistics and Bioinformatics Core, Karmanos Cancer Institute, Detroit, MI 48201, USA; fy7392@wayne.edu; 2 Department of Oncology, Wayne State University School of Medicine, Detroit, MI 48201, USA; lijing@wayne.edu; 3 Pharmacology and Metabolomics Core, Karmanos Cancer Institute, Detroit, MI 48201, USA; 4 Department of Chemistry, University of Louisville, Louisville, KY 40292, USA; xiang.zhang@louisville.edu

**Keywords:** compound identification, differential evolution, gas chromatography–mass spectrometry, metaheuristic algorithms, optimization

## Abstract

**Highlights:**

**What are the main findings?**

• Differential evolution (DE) tuned spectral preprocessing parameters without predefined search spaces.
• Even within predefined parameter spaces, DE achieved modestly improved identification performance relative to grid search.
• DE provides a data-driven approach for exploring unknown or high-dimensional parameter spaces without requiring predefined discretization.
• The Julia-based command line tool MSTune implements both DE and grid search for flexible parameter tuning.

**Abstract:**

**Background/Objectives**: In gas chromatography–mass spectrometry (GC-MS) library-based compound identification, spectrum preprocessing and associated tuning parameters critically influence identification performance. These parameters are conventionally optimized using grid search, which requires predefined parameter spaces and becomes computationally inefficient as dimensionality increases, often failing to identify optimal values because of discretization. Differential evolution (DE), a population-based metaheuristic optimization algorithm, provides a flexible alternative through efficient global exploration of the parameter space. This study compared the performance of DE and grid search for optimizing compound identification. **Methods**: Cosine similarity was applied to the NIST GC-MS library. DE was used to maximize either cross-validated accuracy or mean reciprocal rank (MRR). Results were compared with those from a grid search over five equally spaced parameter values. Identification performance was evaluated using accuracy, MRR, and area under the receiver operating characteristic curve (AUC). **Results**: When all four parameters were optimized simultaneously, DE achieved slightly higher cross-validated accuracy and MRR than grid search, although the absolute differences were modest. More pronounced differences were observed in specific unidimensional tuning scenarios, particularly for the intensity weight factor. Simultaneous multidimensional parameter optimization yielded better performance than isolated parameter tuning. **Conclusions**: Grid search may be computationally advantageous when the parameter space is known and limited, whereas DE provides a more flexible approach for unknown or high-dimensional search spaces. Overall, DE achieved comparable identification performance to grid search, with modest improvements observed in some optimization settings. A command line Julia-based tool, MSTune, was developed for spectrum preprocessing parameter optimization and is publicly available on GitHub.

## 1. Introduction

Gas chromatography–mass spectrometry (GC-MS) is a widely used analytical platform for analysis of small molecules in metabolomics [1,2,3,4]. Its utility is largely driven by reproducible electron ionization fragmentation and the availability of comprehensive spectral libraries, which enable standardized, library-based identification approaches. In this setting, each query spectrum is compared against a collection of reference spectra with known identities. Here, a similarity score is computed for each comparison, and the identity is assigned according to the highest scoring match.

Despite numerous methodological developments, accurate compound identification remains a persistent challenge [5,6,7,8]. Biological samples are chemically complex and often contain co-eluting species and structurally related compounds that generate overlapping or ambiguous spectral features [6,7,9,10,11,12]. Consequently, identification performance depends not only on the quality and coverage of the reference library but also critically on the preprocessing and representation of spectra prior to similarity evaluation [5,13,14]. Typical spectrum preprocessing procedures involve multiple steps, such as noise filtering, peak selection, intensity transformation, and normalization. In particular, noise removal, entropy-based filtering, and intensity weighting introduce multiple tunable parameters that collectively determine the effective representation of the spectra [5,10,15], and small changes in these parameters can substantially affect identification accuracy.

Parameter tuning is commonly performed using heuristic rules or grid search over predefined parameter ranges. Although straightforward to implement, such approaches are inherently limited in their ability to explore high-dimensional parameter spaces and may fail to identify optimal configurations due to the discretization of the parameter space. When prior information is unavailable or the predefined parameter range is poorly specified or does not include the true optimum, grid search may produce unstable or suboptimal solutions. In particular, when the parameter space becomes high dimensional with wide ranges, a grid search becomes a combinatorial problem and can become computationally infeasible [16]. Furthermore, the optimal values for the tuning parameters are not universal but are data dependent and may vary across datasets [17]. Thus, it is recommended to tune parameters at least when the reference library is updated. The relationship between these parameters and identification performance is generally non-smooth, non-convex, and non-differentiable, and the corresponding objective function defined by identification accuracy or ranking-based metrics shares these properties, which implies that gradient-based optimization methods are not well suited for this problem. However, a systematic and computationally efficient framework for optimizing these preprocessing parameters in GC-MS library-based identification remains underdeveloped.

Metaheuristic optimization algorithms provide a flexible alternative for optimizing such complex and non-differentiable objective functions. These optimization methods are designed to explore complex and multimodal landscapes without reliance on derivative information and have been successfully applied in a range of biological and medical applications, including pharmacokinetic modeling [18], magnetic resonance image analysis [19], and electromyographic signal analysis [20], among many others. Differential evolution (DE) [21] is a widely used metaheuristic optimization algorithm [22] that iteratively improves candidate solutions through mutation, recombination, and selection. It has demonstrated strong empirical performance in continuous, high-dimensional optimization settings, particularly when objective evaluations are computationally expensive and the objective surface lacks smooth structure [22]. Numerous optimization approaches have been proposed for parameter tuning, including genetic algorithms [23], particle swarm optimization [24], simulated annealing [25], Bayesian optimization [26], and differential evolution [21]. Among these, DE has been widely applied to continuous optimization problems because of its relative simplicity, robustness, and ability to explore complex parameter spaces.

In this work, we apply DE for parameter tuning to address the limitations of grid search, including the requirement for predefined parameter ranges and computational burden. We then introduce MSTune, a command line Julia-based package for optimizing spectrum preprocessing parameters in mass spectrometry library-matching frameworks. Although the present study focuses on GC-MS applications, the MSTune framework is generally applicable to both GC-MS and liquid chromatography–mass spectrometry (LC-MS) workflows. MSTune is used to maximize the cross-validated identification performance, quantified using metrics such as accuracy, mean reciprocal rank (MRR), and, as a supplementary evaluation metric, area under the receiver operating characteristic curve (AUC). By formalizing parameter tuning as an optimization problem, this work provides a framework for improving library-based compound identification in mass spectrometry while also contributing to the open-source Julia ecosystem of tools used in the analysis of mass spectrometry data.

## 2. Materials and Methods

### 2.1. Data Description

The query mass spectra were obtained from the repetitive library of the NIST 08 Mass Spectral Library (NIST08/2008), and the reference mass spectra were extracted from the NIST Chemistry WebBook [27]. Note that the multiple mass spectra for the same compound in the repetitive library were generated from different experiments. Taking the intersection of these two collections of mass spectra yielded 12,850 unique query compounds, some of which were repeated for a total of 21,516 query spectra. The reference library consisted of 23,721 compounds, each with exactly one spectrum. The mass/charge values ranged from 1 to 892. No compound family-based filtering was performed, and the analyzed spectra represent a broad subset of compounds contained within the NIST resources used in this study. This query and reference library are in line with our previous work [5] and are publicly available on Zenodo (https://zenodo.org/records/12786324accessed on 7 May 2026) [28].

### 2.2. Spectrum Preprocessing Transformations

Three spectrum preprocessing transformations are considered:

○ Weight factor transformation: Given a pair of user-defined weight factor parameters $\left(a,b\right)$ and spectrum I with mass/charge values $\left(m_{1},m_{2},\ldots ,m_{n}\right)$ and intensities $\left(x_{1},x_{2},\ldots ,x_{n}\right)$, the transformed spectrum $I^{⋆}$ has the same mass/charge values as I and has intensities given by $I^{⋆}=\left(m_{1}^{a}\cdot x_{1}^{b},m_{2}^{a}\cdot x_{2}^{b},\ldots ,m_{n}^{a}\cdot x_{n}^{b}\right)$ [10].
○ Noise removal: Given a user-defined noise removal threshold $X\in \left[0,\;1\right]$, all ion fragments with intensity less than X⋅(max intensity in spectrum) are removed.
○ Low-entropy transformation: Given the normalized intensities of a mass spectrum $I=\left(x_{1},\ldots ,x_{n}\right)$ with $\sum_{i=1}^{n}x_{i}=1$, the low-entropy transformation is applied to obtain $I^{⋆}=(x_{1}^{⋆},\ldots ,x_{n}^{⋆})$ with the following:

$$x_{i}^{*}=\left\{\begin{array}{ll} x_{i}, & H_{S}\left(I\right)\geq T\\ x_{i}^{0.25\left(1+H_{S}\left(I\right)\right)}, & H_{S}\left(I\right)<T \end{array}\right.,$$

where T is a user-defined nonnegative real number, $H_{S}(I)$ is the Shannon entropy of I, and i=1,…,n [15].

### 2.3. Similarity Score Computation

Given query spectrum intensities $I=(x_{1},\ldots ,x_{n})$ and reference spectrum intensities $J=(y_{1},\ldots ,y_{n})$, the cosine similarity is computed as follows:

$$S_{C}\left(I,J\right)=\frac{I\circ J}{|I{|}_{2}\cdot |J{|}_{2}}$$

where multiplication in the numerator refers to the dot product of I, and J and multiplication in the denominator refers to scalar multiplication of the $l^{2}$-norm of I and the $l^{2}$-norm of J. To speed up computations, the matrix of query spectra Q and the matrix of reference spectra R, each with rows representing individual spectra and columns representing mass/charge values, was constructed. The weight factor transformation was applied to each row, and this was followed by the low-entropy transformation. Each row was then $l^{2}$ normalized, and then the matrix multiplication $QR^{T}$ was performed to generate similarity scores.

### 2.4. Optimization Approaches to Parameter Tuning

The parameter set consisted of four parameters: one low-entropy threshold, one noise removal threshold, and two weight factors (a, b). The two-optimization metrics considered were accuracy and MRR. Parameter tuning was performed using five-fold cross-validation. The query dataset was partitioned into five equal-sized folds. Each fold was used once as the test dataset, while the remaining four folds were concatenated to form the training dataset. Cross-validation folds were generated at the spectrum level. Thus, replicate spectra corresponding to the same compound may have appeared in multiple folds. For each parameter configuration, the metric was computed on the training dataset and subsequently evaluated on the corresponding test fold. The objective function took the four parameters as input and returned the minimum metric value across the five test folds. The more conservative minimum metric value across the five test folds was used rather than the mean to identify parameter configurations demonstrating relatively stable performance across a wider variety of partitions, thereby reducing sensitivity to easier data partitions [29]. Although the mean is more conventional, the minimum-based objective emphasizes robustness under worst-performing fold conditions [30].

Differential evolution [21] with a population size of 50 and 5000 iterations was used to identify parameter sets that maximize the objective function. Determination of population size remains non-trivial, and a general guideline may be 50 to 10 × (number of parameters being optimized) [31]. Based on empirical observations, 5000 iterations were used regardless of any other stopping criterion because both objective functions and parameter estimates had largely plateaued by iteration 5000. In addition, default stopping criteria involve a magnitude of change in objective function below some threshold for 10,000 iterations. A binomial crossover strategy was used. A grid constructed from 5 equally spaced points from each parameter was constructed, and the objective for each parameter set in the grid was evaluated. Both the multidimensional case of tuning all four parameters simultaneously and the unidimensional case of tuning each individual parameter separately while holding the other three fixed at their natural values (i.e., weight factor *m*/*z* of 0, weight factor intensity of 1, low-entropy threshold of 0, and noise removal threshold of 0) was done. The range of permissible parameter values was liberally set to be [0.0, 5.0] for the three non-noise removal parameters and [0.0, 1.0] for the noise removal parameter. Once optimal parameters were identified for each optimization configuration, the corresponding similarity scores and identification results were computed. In addition, 95% confidence intervals for differential evolution parameter estimates were computed using the bootstrap method with 1000 resampling of the query library.

### 2.5. Brief Software Overview

The Julia-based command line tool MSTune was developed for tuning spectrum preprocessing parameters to maximize a cross-validated metric of either accuracy or MRR. Because DE incurs greater computational costs than grid search, efficient implementation is important. Julia is widely recognized as a high-performance programming language [31], and its speed, usability, and expanding ecosystem of packages have contributed to its increasing adoption in the biological sciences [32]. To minimize the computational burden associated with DE-based parameter optimization in compound identification, MSTune was implemented in Julia. DE was implemented using BlackBoxOptim.jl in Julia with the de_rand_1_bin method, corresponding to a DE/rand/1 mutation strategy with binomial crossover. The population size was set to 50, and the maximum number of optimization steps was set to 5000. Parameter bounds were specified using SearchRange, and the dimensionality was set to the number of optimized parameters. Random number generation was controlled using a Mersenne Twister initialized with the specified random seed. Parameters not explicitly specified, including crossover rate and mutation scaling settings, used the default values in BlackBoxOptim.jl.

MSTune provides three primary functionalities:

1. Tune parameters to maximize cross-validated accuracy or cross-validated MRR using differential evolution optimization.
2. Compute cross-validated accuracy or cross-validated MRR for all parameter combinations in a user-defined grid.
3. Compute similarity scores between all query spectra and reference spectra for a single user-defined parameter set.

Complete usage instructions for MSTune are available on GitHub: https://github.com/hdlugas/MSTune(accessed on 7 May 2026).

### 2.6. Evaluation Metrics

For a given set of optimal parameters, the similarity scores and identification results are recorded. From these data, three evaluation metrics are computed:

○ Accuracy:

$$Accuracy=\frac{number\;of\;query\;spectra\;correctly\;identified}{total\;number\;of\;query\;spectra}$$

○ Mean reciprocal rank (MRR):
  $$MRR=\frac{1}{\left|Q\right|}\sum_{i=1}^{\left|Q\right|}\frac{1}{rank_{i}}\;,$$
  where $\left|Q\right|$ denotes the total number of query spectra, and $rank_{i}$ denotes the rank of the true compound in the ranked list of predicted compounds for query spectrum i.

○ Area under the receiver operating characteristic curve (AUC):
  For each query spectrum, the similarity score of the highest ranked candidate was used as a continuous ranking statistic for ROC analysis because practical library-based compound identification is based on the top-ranked match. The class label indicated whether the top-ranked candidate corresponded to the correct compound. This query-level formulation was chosen because the objective of library searching is compound identification rather than discrimination among all possible query reference pairs. This approach is equivalent to that used previously [5].

Global sensitivity analysis was conducted using the Morris method [33]. The mean absolute elementary effect $\mu_{k}^{⋆}$ and the standard deviation of elementary effects $\sigma_{k}$ were defined as follows:

$$\mu_{k}^{⋆}=\frac{1}{r}\sum_{i=1}^{r}|EE_{k}\left({\vec{x}}_{i}\right)|,$$

$$\sigma_{k}=\sqrt{\frac{1}{r}\sum_{i=1}^{r}{\left({EE}_{k}\left({\vec{x}}_{i}\right)-\mu_{k}\right)}^{2},}$$

where

$$\mu_{k}=\frac{1}{r}\sum_{i=1}^{r}{EE}_{k}\left({\vec{x}}_{i}\right)$$

is the mean elementary effect of the k-th parameter,

$${EE}_{k}\left(\vec{x}\right)=\frac{f\left(\vec{x}+\Delta {\vec{e}}_{k}\right)-f\left(\vec{x}\right)}{\Delta }$$

is the elementary effect of the k-th parameter, r=150 denotes the number of trajectories, ${\vec{x}}_{i}$ values that were equally spaced over the parameter domain, Δ denotes the default adaptive step width, and ${\vec{e}}_{k}$ is the unit vector in the direction of the k-th axis. The overall influence of parameter k is reflected by $\mu_{k}^{⋆}$, while $\sigma_{k}$ indicates nonlinearity and interaction effects associated with parameter k.

## 3. Results

### 3.1. DE Convergence

**Figure 1:**
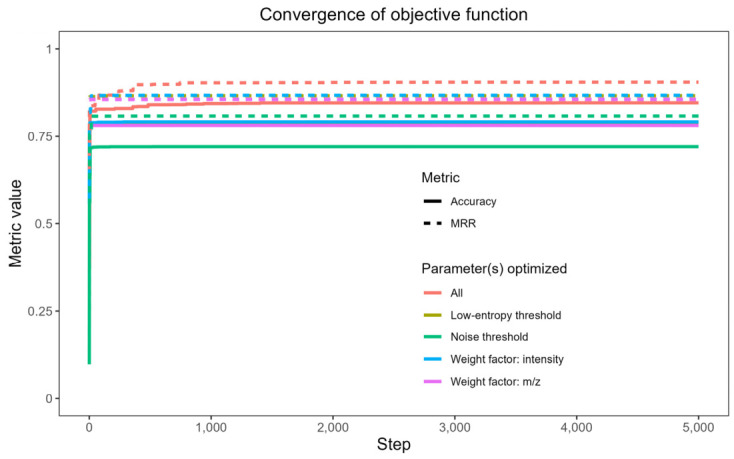
Convergence of objective function in DE optimization for each optimization configuration. MRR: mean reciprocal rank.

DE demonstrated stable convergence across all optimization configurations as observed in Figure 1. Even when all four parameters were tuned simultaneously, the objective function increased rapidly during early iterations and plateaued, indicating adequate exploration of the parameter space.

**Figure 2:**
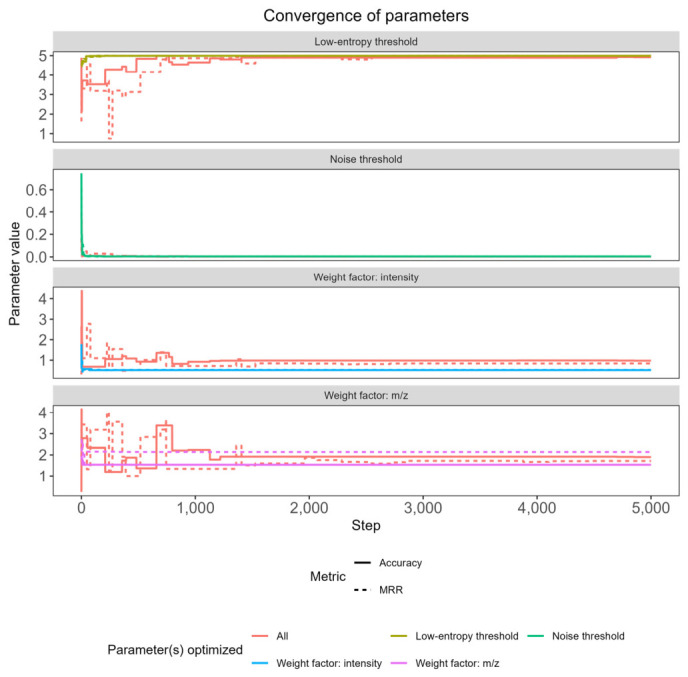
Convergence of parameters in DE optimization across different optimization configurations. MRR: mean reciprocal rank.

As observed in Figure 2, parameter convergence revealed that the low-entropy threshold consistently converged near the upper boundary of the search interval, suggesting that a non-trivial low-entropy transformation improves compound identification. The noise removal threshold and intensity weight factor parameters converged near 0, indicating limited benefit from additional filtering or intensity weighting. The *m*/*z* weight factor converged near 0 in the unidimensional case and around 1.8 in the multidimensional case. Regardless of the metric used, the weight factor parameters in unidimensional optimization converged to slightly different values than their corresponding optimal values from multidimensional optimization, illustrating interaction effects among parameters.

### 3.2. Optimal Parameter Estimates

The optimal parameter values obtained from both DE and the grid search are summarized in Table 1.

When optimizing all four parameters simultaneously, DE achieved a maximum cross-validated accuracy of 0.846 compared to 0.838 obtained using grid search. For cross-validated MRR optimization, DE attained 0.904 compared to 0.898 for the grid search in the case of all four parameters being optimized together. For both metrics, DE achieved slightly higher objective function values than grid search, although the absolute differences were small.

The percent change in cross-validated accuracy between DE and the grid search was $\frac{0.846-0.838}{0.838}\cdot 100=0.959\%$ for the case of all four parameters being optimized simultaneously. This percent change is 0.127%, 0.139%, 19.637%, and 0.256% for the cases of unidimensional tuning of the low-entropy threshold, noise threshold, intensity weight factor, and mass/charge weight factor for the cross-validated accuracy metric, respectively. With respect to the cross-validated MRR metric, the percent change between DE and the grid search was 0.668%, 0.0%, 0.124%, 14.531%, and 0.234% for the cases of all parameters being optimized simultaneously and the four unidimensional cases of low-entropy threshold, noise threshold, intensity weight factor, and mass/charge weight factor, respectively. When only the intensity weight factor was tuned unidimensionally, DE achieved substantially higher objective function values than grid search; however, this observation may reflect coarse grid discretization rather than intrinsic superiority of DE. Finer grid resolution may reduce this observed discrepancy, although at the cost of substantially more objective function evaluations. Additionally, when cross-validated MRR is used as the metric, the percent change between DE and the grid search tends to be smaller compared to when cross-validated accuracy is used as the metric. Bootstrap confidence intervals were evaluated but are not reported for the grid search parameter estimates. Because grid search selects optima from a finite set of discrete candidate values, the resulting bootstrap distributions were often highly discrete or degenerate, making conventional percentile-based confidence intervals difficult to interpret. Similar limitations of bootstrap percentile intervals for discrete or non-smooth estimators have been discussed previously [34,35].

### 3.3. ROC Curve Analysis

**Figure 3:**
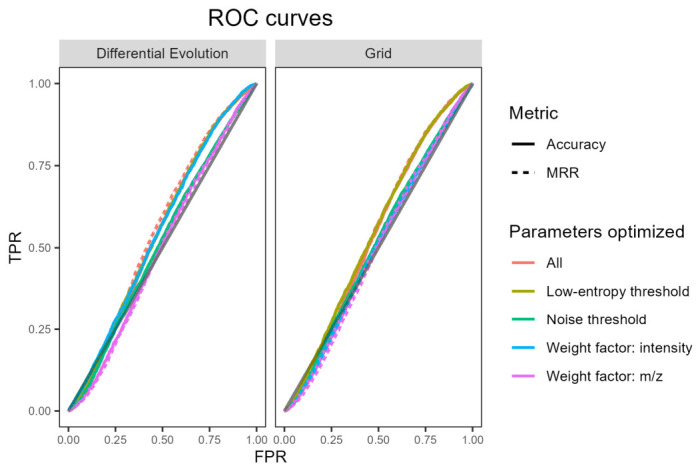
ROC curves for each optimization configuration. TPR: true positive rate; FPR: false positive rate; MRR: mean reciprocal rank.

The ROC curves are shown in Figure 3, and the corresponding AUC values are reported in Table 2. Because practical library search workflows assign a single top-ranked candidate to each query spectrum, ROC analyses were based on top-match similarity scores rather than all query reference pairs. With multidimensional optimization, DE attained an AUC [95% confidence interval] of 0.543 [0.531, 0.554] with cross-validated accuracy as the metric. This confidence interval overlaps with the grid-based counterpart of [0.528, 0.551], as do all other confidence intervals comparing the cross-validated accuracy with cross-validated MRR as the metric. Because the primary goal of library-based compound identification is accurate ranking rather than binary classification, ROC/AUC analysis was considered a supplementary evaluation metric. The relatively low AUC values indicate limited discrimination when the top-match similarity score is interpreted as a binary classification score, whereas rank-based metrics such as rank 1 accuracy and MRR provide a more direct assessment of identification performance.

### 3.4. Ranked Identification Accuracy

The rank N identification accuracies of the optimal parameters applied to the entire query dataset are reported in Table A1 of the Appendix. When all parameters were optimized via DE with the cross-validated accuracy metric, the rank 1 accuracy and 95% confidence interval was 0.849 [0.844, 0.854], increasing to 0.990 [0.989, 0.991] by rank 10. Similar improvements were observed with cross-validated MRR as the metric. McNemar’s test (without continuity correction) indicated that the rank 1 identification accuracies obtained using the optimal parameter sets from multidimensional DE and grid search differed significantly (*p* < 0.001). However, the absolute improvement in rank 1 accuracy was modest (0.849 vs. 0.844).

Differences between DE and the grid search became more pronounced in the unidimensional optimization setting. For example, optimizing only the intensity weight factor using grid search resulted in a substantially lower rank 1 accuracy compared to DE (0.672 [0.666, 0.679] compared to 0.798 [0.792, 0.803]), possibly indicating that either a finer discretization or a more sophisticated optimization approach such as DE is required.

### 3.5. Global Sensitivity Analysis

**Figure 4:**
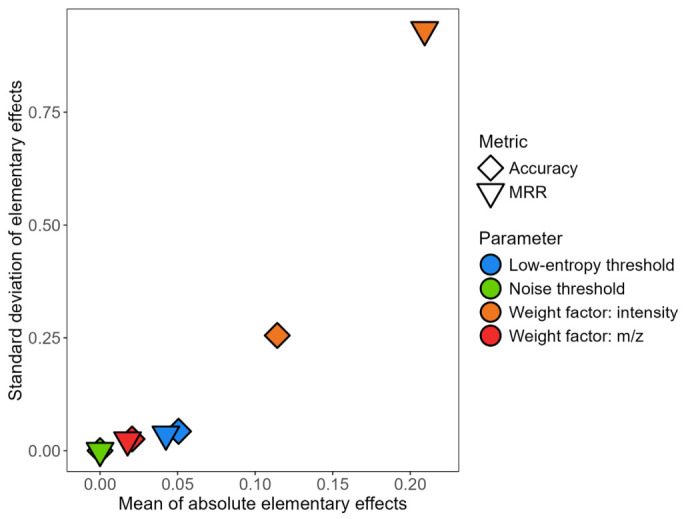
Morris global sensitivity analysis. For each parameter, the mean absolute elementary effect is plotted against the standard deviation of the elementary effects. A larger mean absolute elementary effect indicates greater overall parameter influence, whereas a larger standard deviation of the elementary effects indicates a greater degree of nonlinearity or interaction effects.

The Morris sensitivity analysis results are shown in Figure 4. The intensity weight factor parameter exhibited both the largest mean absolute elementary effect and the largest standard deviation of the elementary effects, indicating both strong overall influence and a high degree of nonlinearity and interaction effects with the other parameters. This observation indicates that the intensity weight factor may have a more complex effect on overall performance compared to other similarity measures. Similar trends in parameter influence were observed regardless of whether accuracy or MRR was maximized.

## 4. Discussion

In this study, we formulated spectrum preprocessing parameter selection in GC-MS compound identification as an optimization problem and applied DE to maximize identification performance. Across all evaluated configurations, DE achieved performance comparable to that of grid search and produced modest improvements in several optimization settings, particularly when all spectrum preprocessing parameters were optimized simultaneously. These findings suggest that metaheuristic optimization provides a practical alternative to coarse, user-defined grid tuning. In particular, DE is less dependent on prior specification of reasonable parameter values and enables data-driven exploration of the parameter space.

Computationally, DE required more objective function evaluations than the grid search. However, the average time required per objective function evaluation was only slightly higher for DE than for grid search (0.475 vs. 0.422 min per evaluation, respectively), indicating that the computational cost of an individual objective function evaluation was similar for the two approaches. For the four-parameter problem considered here, this additional cost was manageable. However, unlike grid search, DE does not rely on exhaustive enumeration of discretized parameter combinations, and its computational burden does not increase combinatorially with the dimensionality, resolution, or range of parameter space. As the dimensionality and complexity of the parameter space increases, grid search rapidly becomes computationally infeasible because the number of parameter combinations grows exponentially [16]. In contrast, DE has been reported to perform well in higher-dimensional or poorly specified parameter spaces, although the present study evaluated only a low-dimensional optimization problem involving one or four tuning parameters. In practice, DE is particularly advantageous when the parameter space is high-dimensional or difficult to specify a priori, whereas grid search may remain suitable for low-dimensional problems with well-defined parameter ranges. Parallel or GPU-based implementations may accelerate both DE and grid search. However, even with parallelization, grid search remains fundamentally limited by the combinatorial growth in the number of parameter combinations as dimensionality increases.

Although DE was used in this study, other population-based metaheuristic optimization algorithms, such as genetic algorithms [36], particle swarm optimization [37], and cross-entropy optimization methods [38] could also be applied to this problem. These methods stochastically explore the parameter space without requiring discretization and are generally more flexible than grid search, particularly in higher-dimensional settings. However, when the parameter space is small and prior information is accurate, grid search may remain computationally efficient and competitive. Although the computational cost of DE increases with the number of function evaluations, DE does not rely on exhaustive enumeration of the parameter space and therefore could be more scalable in practice compared to a grid search with a fine discretization. A key advantage of DE is that it does not require precise prior specification of parameter ranges and can adapt to dataset- or library-dependent characteristics, thereby enabling data-driven parameter tuning.

Several limitations should be noted. First, optimal parameter values and relative performance differences may vary across datasets, especially those generated from different instruments. All analyses were conducted using NIST-derived datasets. Consequently, the generalizability of the optimal parameter values and observed performance characteristics to other instruments, laboratories, or spectral libraries remains uncertain. Future studies should evaluate MSTune using independent external datasets to assess the robustness and transferability of the proposed optimization framework. Relatedly, although the MSTune framework is extensible to LC-MS workflows, no LC-MS datasets were evaluated in the present study. Therefore, applicability to LC-MS remains to be established through future empirical validation. Second, because of the computational burden introduced by more complex similarity measures, only cosine similarity was considered in this study. In addition to its computational efficiency, cosine similarity was selected because it is widely used in mass spectral library matching and enables efficient computation of pairwise similarity scores. However, the optimal preprocessing parameters may depend on the selected similarity metric. Future studies should evaluate MSTune using alternative similarity measures to assess the robustness of the observed findings across different spectral-matching frameworks. Third, several aspects of the parameter-space specification may have influenced the resulting optimal parameter estimates. The estimated optimal low-entropy threshold was consistently at or near the upper boundary of the search interval. The upper bound of five was selected for consistency with Li et al. [6]; however, the repeated convergence near this boundary suggests that the optimal value may lie beyond the investigated range. More generally, the parameter search ranges were selected to be consistent with values previously investigated in the literature [5,15] and to provide a common search domain for both DE and grid search optimization. Because the present study focused on comparing optimization strategies rather than identifying universally optimal parameter ranges, the sensitivity of the results to alternative search boundaries was not formally evaluated. Future studies focused on maximizing identification performance should explore broader parameter ranges and assess the sensitivity of results to the choice of permissible parameter values. Additionally, the grid resolution was intentionally coarse to reflect grid search procedures commonly used in practice. Although a finer grid may reduce performance differences, particularly in the unidimensional intensity weight factor analysis, it would substantially increase computational cost and would be less representative of typical grid search implementations in compound identification studies [5,15]. Fourth, the present comparison was designed to evaluate DE relative to a coarse grid search strategy representative of common practice rather than under a matched computational budget. Future studies should investigate the relative efficiency of DE and grid search under comparable numbers of objective function evaluations. Lastly, another methodological consideration is the choice of optimization objective during cross-validation. In this study, parameter tuning was based on the minimum performance across the five validation folds rather than the more commonly used mean performance. This robustness-oriented criterion was intentionally selected to favor parameter configurations that maintain stable performance across varying data partitions and to reduce sensitivity to parameter sets that perform well primarily on easier subsets of the data. Although optimization based on mean cross-validated performance may yield different optimal parameter values, the minimum-based objective was chosen to prioritize robustness under worst-performing fold conditions. Future studies may investigate the sensitivity of the resulting optimal parameters and identification performance to alternative aggregation criteria, including mean cross-validated performance.

The present study represents a relatively low-dimensional optimization problem involving either one or four tuning parameters. Under these conditions, DE produced only slight improvements relative to a coarse grid search. Nevertheless, DE provides flexibility by avoiding discretization of parameter space and may offer practical advantages as dimensionality, parameter interactions, or uncertainty regarding appropriate parameter ranges increase; however, this hypothesis was not directly evaluated in the present study and requires future empirical investigation. Because the analyses were not performed under matched computational budgets, the present results should be interpreted as a comparison of DE to current parameter optimization approaches rather than as a definitive efficiency benchmark between optimization strategies.

Overall, these results indicate that DE provides a systematic and flexible approach for tuning spectrum preprocessing parameters. In the present GC-MS dataset, performance gains over grid search were modest in the multidimensional setting but more apparent in specific unidimensional cases. Although DE frequently achieved higher performance metrics than grid search, many of the observed differences were numerically small, and several confidence intervals overlapped substantially. Therefore, the results should be interpreted primarily as evidence that DE provides a viable and flexible optimization strategy rather than as evidence of large performance gains over grid search. The relatively low dimensionality of the parameter space and the coarse discretization used in the grid search may have limited the potential differences between optimization strategies. Future studies involving more complex parameter spaces may help clarify whether larger performance differences emerge. While the present study focused on GC-MS library-based identification, the proposed MSTune framework is generally applicable to both GC-MS and LC-MS workflows. In particular, LC-MS preprocessing and spectral matching pipelines often involve larger and more complex parameter spaces, including retention-time and window size-related tuning parameters, for which exhaustive grid-based optimization may become increasingly impractical. In such settings, DE-based optimization may provide practical advantages through efficient exploration of parameter space, although the magnitude of any resulting performance improvements remains to be determined.

## Figures and Tables

**Table 1:** Optimal parameters from each optimization configuration.

Optimization	Metric	Parameters Optimized	Low-Entropy Threshold	Noise Threshold	Weight Factor: Intensity	Weight Factor: *m*/*z*	Max Value of Metric
DE	Accuracy	All	4.924 [4.448, 4.998]	0.003 [0.003, 0.007]	0.975 [0.798, 1.161]	1.901 [1.677, 2.696]	0.846
		LE threshold	4.995 [4.933, 5.000]	0	1	0	0.790
		Noise threshold	0	0.005 [0.002, 0.012]	1	0	0.720
		WF: intensity	0	0	0.514 [0.501, 0.583]	0	0.792
		WF: mass/charge	0	0	1	1.536 [1.423, 2.298]	0.782
	MRR	All	4.985 [4.394, 5.000]	0.003 [0.000, 0.007]	0.841 [0.794, 1.250]	1.717 [1.464, 2.485]	0.904
		LE threshold	4.995 [4.991, 5.000]	0	1	0	0.865
		Noise threshold	0	0.005 [0.003, 0.012]	1	0	0.809
		WF: intensity	0	0	0.524 [0.501, 0.574]	0	0.867
		WF: mass/charge	0	0	1	2.140 [1.545, 2.298]	0.856
Grid	Accuracy	All	5	0	1.25	2.5	0.838
		LE threshold	5	0	1	0	0.789
		Noise threshold	0	0	1	0	0.719
		WF: intensity	0	0	1.25	0	0.662
		WF: mass/charge	0	0	1	1.25	0.780
	MRR	All	5	0	1.25	3.75	0.898
		LE threshold	5	0	1	0	0.865
		Noise threshold	0	0	1	0	0.808
		WF: intensity	0	0	1.25	0	0.757
		WF: mass/charge	0	0	1	2.5	0.854

DE: differential evolution; MRR: mean reciprocal rank; LE: low entropy; WF: weight factor. Values in brackets indicate 95% confidence intervals.

**Table 2:** AUC across various optimization configurations.

Optimization	Metric	Parameters Optimized	AUC [95% CI]
DE	Accuracy	All	0.543 [0.531, 0.554]
		LE threshold	0.550 [0.539, 0.560]
		Noise threshold	0.513 [0.504, 0.522]
		WF: *m*/*z*	0.550 [0.540, 0.561]
		WF: intensity	0.499 [0.489, 0.509]
	MRR	All	0.558 [0.546, 0.569]
		LE threshold	0.550 [0.539, 0.560]
		Noise threshold	0.513 [0.504, 0.522]
		WF: *m*/*z*	0.549 [0.539, 0.559]
		WF: intensity	0.493 [0.483, 0.503]
Grid	Accuracy	All	0.539 [0.528, 0.551]
		LE threshold	0.550 [0.540, 0.560]
		Noise threshold	0.513 [0.504, 0.522]
		WF: *m*/*z*	0.501 [0.493, 0.510]
		WF: intensity	0.504 [0.494, 0.514]
	MRR	All	0.540 [0.529, 0.552]
		LE threshold	0.550 [0.540, 0.560]
		Noise threshold	0.513 [0.504, 0.522]
		WF: *m*/*z*	0.501 [0.493, 0.510]
		WF: intensity	0.491 [0.481, 0.500]

CI: confidence interval; DE: differential evolution; MRR: mean reciprocal rank; LE: low entropy; WF: weight factor; AUC: area under (Receiver Operator Characteristic) curve.

## Data Availability

The gas chromatography–mass spectrometry data used in this study are publicly available on Zenodo (https://zenodo.org/records/12786324) [X]. The Julia-based command line tool MSTune used to identify optimal parameters using (i) DE and (ii) exhaustive grid search is publicly available on GitHub (https://github.com/hdlugas/MSTune).

## Abbreviations

The following abbreviations are used in this manuscript:

DE	Differential evolution
FPR	False positive rate
GC-MS	Gas chromatography–mass spectrometry
LC-MS	Liquid chromatography–mass spectrometry
MRR	Mean reciprocal rank
ROC	Receiver operator characteristic
TPR	True positive rate

## Appendix A

**Table A1:** Ranked outcomes with optimal parameters. A rank N identification is considered to be true if the true compound has one of the N largest similarity scores with the given query spectrum. Note that these outcomes are with respect to optimal parameters applied to the entire query dataset.

Optimization	Metric	Parameters Optimized	Rank	Accuracy
DE	Accuracy	All	1	0.849 [0.844, 0.854]
			2	0.934 [0.930, 0.937]
			3	0.963 [0.960, 0.965]
			4	0.973 [0.971, 0.975]
			5	0.980 [0.978, 0.982]
			10	0.990 [0.989, 0.991]
		LE threshold	1	0.801 [0.796, 0.806]
			2	0.904 [0.900, 0.908]
			3	0.941 [0.938, 0.944]
			4	0.957 [0.954, 0.960]
			5	0.967 [0.964, 0.969]
			10	0.981 [0.980, 0.983]
		Noise threshold	1	0.728 [0.722, 0.733]
			2	0.838 [0.833, 0.843]
			3	0.884 [0.880, 0.888]
			4	0.908 [0.904, 0.911]
			5	0.921 [0.918, 0.925]
			10	0.949 [0.946, 0.952]
		WF: intensity	1	0.798 [0.792, 0.803]
			2	0.901 [0.897, 0.905]
			3	0.940 [0.937, 0.943]
			4	0.957 [0.954, 0.960]
			5	0.967 [0.965, 0.969]
			10	0.984 [0.982, 0.985]
		WF: mass/charge	1	0.790 [0.784, 0.795]
			2	0.889 [0.885, 0.893]
			3	0.927 [0.923, 0.931]
			4	0.942 [0.939, 0.945]
			5	0.952 [0.950, 0.955]
			10	0.972 [0.969, 0.974]
	MRR	All	1	0.850 [0.844, 0.854]
			2	0.933 [0.930, 0.936]
			3	0.963 [0.960, 0.965]
			4	0.974 [0.971, 0.976]
			5	0.979 [0.978, 0.981]
			10	0.991 [0.990, 0.992]
		LE threshold	1	0.801 [0.796, 0.806]
			2	0.904 [0.900, 0.908]
			3	0.941 [0.937, 0.943]
			4	0.957 [0.954, 0.960]
			5	0.967 [0.964, 0.969]
			10	0.981 [0.980, 0.983]
		Noise threshold	1	0.728 [0.722, 0.734]
			2	0.838 [0.833, 0.843]
			3	0.884 [0.880, 0.888]
			4	0.908 [0.904, 0.912]
			5	0.921 [0.918, 0.925]
			10	0.949 [0.947, 0.952]
		WF: intensity	1	0.798 [0.792, 0.803]
			2	0.901 [0.897, 0.906]
			3	0.941 [0.938, 0.944]
			4	0.957 [0.954, 0.960]
			5	0.967 [0.965, 0.969]
			10	0.983 [0.982, 0.985]
		WF: mass/charge	1	0.790 [0.785, 0.796]
			2	0.889 [0.886, 0.894]
			3	0.928 [0.925, 0.931]
			4	0.943 [0.940, 0.946]
			5	0.953 [0.950, 0.956]
			10	0.972 [0.970, 0.974]
Grid	Accuracy	All	1	0.844 [0.840, 0.849]
			2	0.931 [0.928, 0.934]
			3	0.960 [0.957, 0.963]
			4	0.972 [0.970, 0.974]
			5	0.978 [0.976, 0.980]
			10	0.989 [0.988, 0.990]
		LE threshold	1	0.801 [0.796, 0.806]
			2	0.904 [0.900, 0.908]
			3	0.941 [0.937, 0.944]
			4	0.957 [0.954, 0.960]
			5	0.967 [0.964, 0.969]
			10	0.981 [0.979, 0.983]
		Noise threshold	1	0.727 [0.722, 0.733]
			2	0.838 [0.833, 0.843]
			3	0.883 [0.879, 0.888]
			4	0.907 [0.903, 0.911]
			5	0.922 [0.918, 0.925]
			10	0.949 [0.947, 0.952]
		WF: intensity	1	0.672 [0.666, 0.679]
			2	0.785 [0.780, 0.791]
			3	0.836 [0.831, 0.841]
			4	0.863 [0.858, 0.867]
			5	0.880 [0.876, 0.884]
			10	0.920 [0.916, 0.924]
		WF: mass/charge	1	0.787 [0.782, 0.792]
			2	0.886 [0.882, 0.891]
			3	0.924 [0.921, 0.927]
			4	0.940 [0.937, 0.943]
			5	0.951 [0.948, 0.953]
			10	0.970 [0.968, 0.972]
	MRR	All	1	0.844 [0.839, 0.849]
			2	0.930 [0.927, 0.934]
			3	0.959 [0.956, 0.961]
			4	0.970 [0.968, 0.973]
			5	0.977 [0.975, 0.979]
			10	0.989 [0.987, 0.990]
		LE threshold	1	0.801 [0.795, 0.807]
			2	0.904 [0.900, 0.908]
			3	0.941 [0.937, 0.944]
			4	0.957 [0.954, 0.960]
			5	0.967 [0.964, 0.969]
			10	0.981 [0.979, 0.983]
		Noise threshold	1	0.727 [0.721, 0.733]
			2	0.838 [0.833, 0.843]
			3	0.883 [0.879, 0.888]
			4	0.907 [0.903, 0.911]
			5	0.922 [0.918, 0.925]
			10	0.949 [0.947, 0.952]
		WF: intensity	1	0.672 [0.666, 0.678]
			2	0.785 [0.780, 0.791]
			3	0.836 [0.831, 0.841]
			4	0.863 [0.858, 0.867]
			5	0.880 [0.876, 0.884]
			10	0.920 [0.916, 0.924]
		WF: mass/charge	1	0.787 [0.782, 0.793]
			2	0.889 [0.884, 0.893]
			3	0.927 [0.923, 0.930]
			4	0.943 [0.939, 0.946]
			5	0.953 [0.950, 0.955]
			10	0.972 [0.970, 0.974]

DE: differential evolution; MRR: mean reciprocal rank; LE: low entropy; WF: weight factor.
